# Transporter protein and drug-conjugated gold nanoparticles capable of bypassing the blood-brain barrier

**DOI:** 10.1038/srep25794

**Published:** 2016-05-16

**Authors:** Yanhua Zhang, Janelle Buttry Walker, Zeljka Minic, Fangchao Liu, Harry Goshgarian, Guangzhao Mao

**Affiliations:** 1 Department of Chemical Engineering and Materials Science, Wayne State University, 5050 Anthony Wayne Drive, Detroit, MI 48202, USA; 2 Department of Anatomy and Cell Biology, Wayne State University, 540 E Canfield St, Detroit, MI 48201, USA.; 3 Cardiovascular Research Institute, Wayne State University, 540 E Canfield St, Detroit, MI 48201, USA.

## Abstract

Drug delivery to the central nervous system (CNS) is challenging due to the inability of many drugs to cross the blood-brain barrier (BBB). Here, we show that wheat germ agglutinin horse radish peroxidase (WGA-HRP) chemically conjugated to gold nanoparticles (AuNPs) can be transported to the spinal cord and brainstem following intramuscular injection into the diaphragm of rats. We synthesized and determined the size and chemical composition of a three-part nanoconjugate consisting of WGA-HRP, AuNPs, and drugs for the treatment of diaphragm paralysis associated with high cervical spinal cord injury (SCI). Upon injection into the diaphragm muscle of rats, we show that the nanoconjugate is capable of delivering the drug at a much lower dose than the unconjugated drug injected systemically to effectively induce respiratory recovery in rats following SCI. This study not only demonstrates a promising strategy to deliver drugs to the CNS bypassing the BBB but also contributes a potential nanotherapy for the treatment of respiratory muscle paralysis resulted from cervical SCI.

The leading causes of death among persons with SCI since 1973, according to the National SCI Database, are from pneumonia and septicemia due to damage to respiratory muscle function caused by SCI^1^. The major therapeutic challenge in the treatment of respiratory dysfunction caused by SCI, similar to other neurological diseases, is the inability of many drugs to go across the BBB. The BBB is a natural defense system for the brain and generally only permits diffusion of neutral, lipophilic, and low molecular weight (smaller than 400–600 Da) molecules into the CNS from blood^2^,^3^. WGA is a lectin recognized by *N*-acetylglucosamine and sialic acid receptors located on most neuronal cell membranes^4^,^5^. WGA, in a few studies, has been used to enhance intranasal delivery of peptides to the brain through the olfactory route due to its affinity to the olfactory mucosa^6^,^7^. While the bioconjugation chemistry for chemically attaching the drug and the targeting protein to the AuNP carrier is not new, to our knowledge, this is the first study to use WGA as a targeting moiety to transport drug-bound nanoconjugates exclusively to the phrenic motoneurons that control respiratory function, effectively bypassing the BBB. The size of the nanoconjugates is in the order of tens of nanometers. Here, only WGA functions as the targeting protein while HRP is used historically as a visual reporter. When injected into muscle, WGA-HRP undergoes retrograde transport to the motoneuron cell bodies and is then transsynaptically transported to the pre-motor neurons within 48 h post injection^8^,^9^. We synthesized a three-part nanoconjugate consisting of WGA-HRP chemically bound to AuNPs, which were also chemically bound to drugs previously shown to induce recovery of the diaphragm paralyzed by SCI^10^. Specifically, following intramuscular injection into the diaphragm, the WGA-HRP component of the nanoconjugate engages in receptor-mediated endocytosis resulting in retrograde transport of the nanoconjugate within motoneurons to the phrenic nuclei in the cervical spinal cord and transsynaptically to the rostral ventral respiratory groups (rVRGs) in the medulla. The drug, 1,3-dimethylxanthine (theophylline or THP), is an adenosine receptor antagonist clinically used for the treatment of respiratory dysfunctions such as asthma, bronchitis, and respiratory muscle paralysis resulted from SCI^10^,^11^,^12^. In rats with a spinal cord hemisection at the second cervical spinal segment (C2Hx), systemic (oral or intravenous) administration of THP or another adenosine receptor antagonist, 1,3-dipropyl-8-cyclopentylxanthine (DPCPX), resulted in an increase in respiratory motor output and recovery of the respiratory related activity by stimulating the crossed phrenic pathway (CPP)^10^,^12^. The CPP is a latent pathway, which crosses the midline below the level of SCI injury to synapse on the ipsilateral phrenic nuclei (PN) in the 3^rd^ through 6^th^ segments of the cervical spinal cord while the primary descending pathway, between the neuronal connection from the rVRG in the medulla to the ipsilateral phrenic nucleus, has been severed by a high cervical SCI^8^. However, in humans the therapeutic dose of methylxanthines causes intolerable side effects including nausea and seizures^13^,^14^. These side effects are associated with nonspecific biodistribution and neuronal hyperactivity induced by the drug. Therefore we designed a nanoconjugate in which the drug and the transporter protein are chemically linked through a nanoparticle carrier, the AuNPs, in order to reduce side effects of the drug by delivering it selectively to the respiratory neurons in the cervical spinal cord and medulla. A biodegradable bond between the drug and the nanoparticle carrier enables drug release at the targeted sites. The effectiveness of the nanoconjugate drug delivery was verified by the return of diaphragm muscle function previously paralyzed due to SCI. AuNPs were selected as the drug carrier due to their biocompatibility, tunable colloidal chemistry, and quality control through a wide range of analytical methods^15^,^16^.

Here we focus our description of results on THP while details on DPCPX are described in the Supplementary Information (SI). The nanoconjugate synthesis started with the conversion of THP into its pro-drug, 7-(hydroxymethyl) theophylline or pro-THP, using the Mannich reaction (Fig. 1)^17^. In a typical reaction, 100 mg THP was added to 1 ml 36% aqueous formaldehyde (HCHO), followed by the addition of 1.5 ml triethylamine (Et_3_N). The mixture became homogeneous after vigorous stirring whereupon 2 ml tetrahydrofuran (THF) was added. White solid was collected after stirring for 1 h followed by filtration. The reaction product was analyzed by proton nuclear magnetic resonance (^1^H NMR) in order to verify the formation of pro-THP (SI Fig. 1). The solid was dried and then re-dissolved in 20 ml dimethyl sulfoxide (DMSO) to reach a final concentration of 5 mg/ml of pro-THP. DMSO was used instead of water to avoid hydrolysis of pro-THP back to THP.

In a separate step, AuNPs of 4 nm in core diameter were synthesized by reacting gold chloride (HAuCl_4_) with tri-sodium citrate following the procedure described by Jana *et al*.^17^. After adjusting the pH of the solution to 11 with 0.1 M NaOH, mercaptosuccinic acid (MSA) was added to replace tri-sodium citrate as the capping ligand by forming a strong Au-S bond. After stirring for 12 h at room temperature, the final product, MSA-capped AuNPs, was concentrated to 2.5 mg (gold)/ml by a series of washes with deionized water and ultracentrifugation using 10,000 MW Millipore Amicon^®^ centrifugal filter units.

In the next step, pro-THP was chemically attached to MSA-capped AuNPs to form the drug nanoconjugate, AuNP-pro-THP, via a biodegradable ester bond to enable *in vivo* drug release. For this purpose, the MSA-capped AuNP was concentrated to 5 mg(gold)/ml in 5 ml 0.1 M MES buffer (pH = 4.7) followed by the addition of 1 ml DMSO containing 5.0 mg pro-THP, 5 ml of DMSO containing 4.8 mg 1-ethyl-3-(3-dimethylaminopropyl) carbodiimide (EDC), and 1.7 ml 4-dimethylaminopyridine (DMAP) to start the Steglich reaction^18^. After 40 h, the product AuNP-pro-THP was collected by rinsing with deionized water.

The final nanoconjugate, WGA-HRP-AuNP-pro-THP, was synthesized using carbodiimide chemistry for coupling amines and carboxyls^19^. In order to conjugate WGA-HRP to AuNP-pro-THP through an amide bond, the solution of AuNP-pro-THP was diluted to 0.1 mg/ml by deionized water and was maintained at 4 °C and pH of 6.6 followed by additions of 5 mg WGA-HRP, 10 mg EDC, and 3 mg N-hydroxysuccinimide (NHS) in order to form an amide bond between WGA-HRP and the MSA-capped AuNPs. The reaction proceeded for 1 h and was followed by washing with deionized water. The concentration of the final stock was diluted to approximately 0.8 mg (pro-THP)/ml. The actual drug dosages were determined by chemical analyses as described below.

The nanoconjugates from each stage of synthesis were characterized by UV-*vis* spectroscopy, transmission electron microscopy (TEM), dynamic light scattering (DLS), and thermogravimetric analysis (TGA) (Fig. 2 and SI). The nanoconjugate characterization data for pro-DPCPX can be found in the SI. Figure 2A shows the UV-*vis* spectra of MSA-capped AuNP, AuNP-pro-THP, and WGA-HRP-AuNP-pro-THP obtained in deionized water (pH = 5.8) at room temperature. The UV-*vis* spectra of a constant absorption peak at 508–510 nm are in the expected range for AuNPs with 4 nm core diameter and they also indicate a high degree of colloidal dispersion stability at all stages of the synthesis. Figure 2C–E show TEM images of MSA-capped AuNPs, AuNP-pro-THP, and WGA-HRP-AuNP-pro-THP. The particle size histograms can be found in SI Fig. 3. The AuNPs were well dispersed and the diameter of the AuNPs remained unchanged at 4.1 ± 0.3 nm before and after conjugation with pro-THP according to TEM. The AuNPs size increased slightly to 5.2 ± 1.3 nm. Here, there is uncertainty in particle core size determination due to the conjugated WGA-HRP and overlapping particles. Particle agglomeration, observed in WGA-HRP-AuNP-pro-THP samples, is likely caused by the TEM sample preparation step (Fig. 2D). The Zetasizer provided measurements of the particle hydrodynamic size by DLS and the zeta potential by electrophoresis. The hydrodynamic diameters are 4.8 nm (zeta potential = −28.5 mV) for MSA-capped AuNP, 11.7 nm (zeta potential = −36.6 mV) for AuNP-pro-THP, and 37.8 nm (zeta potential = −35.6 mV) for WGA-HRP-AuNP-pro-THP. The zeta potential measurements were conducted in PBS buffer (pH = 7.4) at 37 °C. The size increase after each conjugation reaction is consistent of successful coupling of the drug or protein to the AuNP. The negative surface charge characteristic of all the nanoparticles provides the necessary electrostatic repulsion to ensure aqueous dispersion stability of the nanoparticles.

The chemical compositions of the intermediate and final nanoconjugates were analyzed by TGA (Fig. 2B). Based on the percentages of weight losses at different stages of synthesis, we were able to estimate that in the final nanoconjugate, there are 204 pro-THP molecules conjugated to one 4 nm MSA-capped AuNP and 2 MSA-capped AuNPs conjugated to one WGA-HRP. The numbers of drug molecules conjugated correspond to 60% occupation for pro-THP of all available carboxyl provided by MSA. The detailed calculations can be found in the SI 1.3. We observed that the number of conjugated drug molecules remained unchanged with increasing drug to Au molar feed ratio in the reaction indicating that a surface saturation was reached possibly due to a steric effect. It is interesting to note that in both THP (Fig. 2B) and DPCPX (SI Fig. 4) cases we obtained similar protein to AuNP molar ratio of 1 to 2. This nanostructure configuration is consistent with the TEM and DLS results.

Three types of nanoconjugates, WGA-HRP-AuNP-pro-THP, WGA-HRP-AuNP-pro-DPCPX, and WGA-HRP-AuNP (control), in PBS buffer (pH = 7.4) were tested by a single time point injection of each nanoconjugate solution into the diaphragm muscle of C2Hx rats. Following a C2Hx, an electromyography (EMG) of the diaphragm muscle was performed to assess the extent of hemidiaphragmatic paralysis. Only C2Hx rats that showed a complete paralysis of the left hemidiaphragm (ipsilateral to C2Hx) received nanoconjugate injections (Fig. 3A). See SI Section 2.3 for dosage details. The dosages used in this study were selected based on a dose response study to determine the lowest dose with maximal recovery. A second EMG was performed in all rats 7 days after the nanoconjugate administration to assess the extent of recovery. All 3 rats in the pro-THP (0.06 mg/kg) nanoconjugate group displayed a return of diaphragm muscle function as displayed in Fig. 3B. In addition, all 3 rats in the pro-DPCPX (0.15 μg/kg) nanoconjugate group displayed a return of diaphragm muscle function as displayed in Fig. 3C. All 3 rats in the control group (injected WGA-HRP-AuNP nanoconjugate) displayed no return of function (Fig. 3D).

Following a C2Hx, 2 additional rats were injected with WGA-HRP-AuNP-pro-THP and were prepared for immunohistochemical analysis of the spinal cord and medulla (a portion of the brainstem). The first incidence of recovery was observed 3 days post injection therefore this time point was selected for immunohistochemical analysis to detect WGA. Three days after injection tissue was removed and treated with anti-WGA antibodies (details in SI Section 2.4). Tissue sections from both rats displayed ipsilateral retrograde labeling of the PN in the spinal cord (cervical 3 to cervical 6) (Fig. 4A–C) and transsynaptic bilateral labeling in the rVRGs in the medulla (Fig. 4D–G). There was no labeling detected any in additional nuclei in the tissue examined similar to past studies using WGA based tracers^8^,^9^. The isolated WGA labeling demonstrates the selective targeting of the nanoconjugate to only the nuclei associated with diaphragm function, the PN and rVRGs. Visualization of WGA along with EMG recordings of the diaphragm suggests the nanoconjugate was successful as a carrier to target drug delivery.

The release of pro-THP from the nanoconjugate was investigated by laser desorption/ionization mass spectrometry (LDI-MS). The nanoconjugate, AuNP-pro-THP, at a concentration of 0.04 mg(Au)/ml was incubated in 20 mM HEPES (4-(2-hydroxyethyl)-1-piperazineethanesulfonic acid) buffer at pH 7.4 or artificial cerebral spinal fluid (ACSF). The ACSF sample was prepared based on the literature procedure^20^. After 12 h incubation, the supernatant was collected by ultracentrifugation using 10,000 MW Millipore Amicon^®^ centrifugal filters and analyzed by LDI-MS. The MS spectra of the supernatants as well as the control (pro-THP dissolved in HEPES) are shown in SI Fig. 5. The supernatants collected from HEPES and ACSF both contain pro-THP after 12 h incubation with the nanoconjugate. We have previously shown that the retrograde transsynaptic transport of WGA-HRP (alone and not conjugated to the nanoconjugate) to both the phrenic nucleus and the medullary respiratory center (rVRG) takes 48 h^8^. Here we show that when WGA-HRP is bound to the drug-coupled AuNPs the first incidence of recovery is observed 72 h following nanoconjugate administration *in vivo*. Thus, we have both chemical and *in vivo* evidence of drug release from the nanoconjugate.

Our work shows that the targeted nanoconjugate induces recovery of respiratory activity in C2Hx rats after a single dose of conjugated THP at 0.06 mg/kg for up to 7 days. In comparison, THP by itself administered in the same surgical model requires a single systemic dose of 15 mg/kg and its effect on respiratory recovery lasts only up to 3 hours^10^. Similarly, the effective dosage of DPCPX conjugated to WGA-HRP-AuNP, 0.15 μg/kg, is much lower than the effective systemic dosage of DPCPX alone, 0.1 mg/kg^12^. Note the nanoconjugate dose is in micrograms whereas the systemic dose is in milligrams. More persistent respiratory recovery was achieved by THP alone but it requires oral administration of THP of 20 mg/kg, 3 times daily, for 3 days^21^. By targeting the drug, the nanoconjugate design has the potential to eliminate unwanted side effects by requiring a smaller effective dose and more persistent recovery after a single injection.

The nanoconjugate described here is unique among other nanotechnology-based drug delivery methods to the brain. The majority of current approaches focus on getting drugs across the BBB by designing the nano-carriers to utilize the three main mechanisms of passive diffusion, carrier-mediated influx, and vesicular transport, particularly through receptor-mediated transport^22^,^23^,^24^. The neurosurgical-based delivery approaches, such as intracerebral implantation, intracerebroventricular infusion, and convection enhanced diffusion, are capable of bypassing the BBB however they are invasive. Transnasal drug delivery to the brain is a non-invasive approach but is limited to delivering lipid-soluble small molecules in the absence of local injury^25^. A few studies have shown that WGA-HRP enables neuropeptides to penetrate the olfactory cerebrospinal fluid (CSF) barrier via absorptive-mediated endocytosis based on WGA binding to membrane lectin sites^8^. Peptide nanoparticles coated with WGA were delivered via the olfactory route^6^. This work takes advantage of the ability of WGA to undergo retrograde transport to the neuron cell bodies for direct access to the CNS, which could overcome the severe limit by the BBB to molecular size, charge, and hydrophilicity. The nanoconjugate chemistry can be further tuned to optimize drug release time and dosage while the nano-carrier size, shape, and surface charge can be varied to enhance cellular uptake by the cell bodies in the CNS. The nanotechnology as described here has the potential to treating respiratory dysfunctions after SCI as well as the treatment of other neurological disorders and brain cancers, which rely on safe and effective delivery of drugs to the brain. The targeted delivery of a nano-carrier has advantages over most of the currently employed methods for treatment of neurological diseases, which are invasive with associated post-surgical complications.

## Conclusions

Here we have shown for the first time a three-part AuNP nanoconjugate engineered to selectively deliver drugs, THP and DPCPX, to a specific population of neurons mediated by WGA uptake. The AuNPs act as a carrier to link the WGA-HRP to the drug via a biodegradable ester bond between the carrier and the drug. Following intramuscular injection the ester bond enables *in vivo* drug release in the cervical spinal cord and medulla nuclei targeted by WGA-HRP. The effect of the drugs is apparent based on the physiological response demonstrated by the return of muscle function to the once paralyzed hemidiaphragm. *In vivo* studies are underway to fully characterize the dose response of both THP and DPCPX versions on the nanoconjugate as well as the short and long term biodistribution and biological effects of the AuNPs. The ability to synthesize a nanoconjugate capable of bypassing the BBB and selectively target a population of neurons opens the door for numerous applications. Injection of WGA-nanoconjugates into select muscles would expose the associated motoneurons to the drug of choice. There is great potential for the application of this engineered nanoconjugate across many neuromuscular disease and injury models.

## Methods

### Materials

Chemicals used in the following experiments: gold(III) chloride trihydrate (HAuCl_4_∙3H_2_O, 99% metal trace); sodium citrate tribasic dehydrate (98%); mercaptosuccinic acid (MSA, 97%); sodium borohydride (NaBH_4_, 98%); 4-dimethylaminopyridine (DMAP, 99%); dimethyl sulfoxide (DMSO, 99.8%); 37% formaldehyde solution; tetrahydrofuran (THF, 99%); N-hydroxysuccinimide (NHS, 98%); dipropylcyclopentylxanthine (DPCPX, 97%); theophylline (THP, 97%) triethylamine (Et_3_N, 99%); lectin from triticum vulgaris (WGA-HRP) (Sigma-Aldrich, St. Louis, MO); 1-Ethyl-3-(3-dimethylaminopropyl) carbodiimide (EDC, 98%) (Sigma-Aldrich, St. Louis, MO).

### Nanoconjugate characterization

UV-*vis* absorption spectroscopy (Varian Cary 50) was used to determine the AuNP size based on the Beer-Lambert law. The scan range was 200–800 nm.

The transmission electron microscope (TEM) images were taken on a JEOL JEM-2010 electron microscope. Samples were prepared by placing a droplet of the nanoconjugate solution on a Formvar-coated copper TEM grid. Excess liquid was removed by filter paper under the grid and the sample was air-dried. The working voltage was 200 keV and the current was 109 mA. For each image, 30 particles were randomly selected and measured to give the nanoparticle size distributions.

The nanoparticle size was determined by dynamic light scattering (DLS) (Zetasizer Nano ZS, Malvern). A one ml solution of nanoparticle in PBS buffer was placed in a 2.0 ml polystyrene cuvette. The backscattering angle Θ was fixed at 180° with a laser wavelength λ = 633 nm. The size measurement range was set between 1 nm and 6 μm.Hydrodynamic diameter (D_H_) is a function of the diffusion coefficient (D), temperature (T), and viscosity (η) according to the Stokes-Einstein equation: 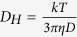, where *k* is the Boltzmann constant, *T* was 37 °C, and *D* was obtained from autocorrelation function via the cumulant fitting. The electrophoretic mobility of the nanoparticles was measured using the laser Doppler velocimetry and phase analysis light scattering technique of the Malvern Zetasizer. The electrophoretic mobility was converted into a zeta potential using the Smoluchowski equation using the Malvern software.

TGA analysis was used to quantify the chemical composition of the nanoconjugate. TGA was performed on the SDT-Q-600 instrument using air as the working gas. The temperature range was 100–800 °C with a heating rate of 10 °C/min.

The LDI-MS measurements were carried out on a Bruker UltrafleXtreme MALDI-TOF/TOF Mass Spectrometer (Lumigen Instrument Center, Detroit). The samples were analyzed by LDI-MS after being transferred onto a MALDI target for LDI-MS analysis without adding any organic matrix. All mass spectra were acquired in the reflection mode and represent an average of 500 laser shots at a repetition frequency at 500 Hz. The accelerating voltage was set to 20 kV. The laser power was optimized in the range of 70–90% for each sample. The Bruker software flexAnalysis (version 3.4) was used for data analysis. Each sample was measured 10 times by LDI-MS.

### Cervical hemisection surgery

All rat studies were approved by the Wayne State University School of Medicine Institutional Animal Care and Use Committee. The following procedures were carried out in accordance with the Code of Ethics of the World Medical Association for experiments involving animals. Eleven adult male Sprague Dawley rats were used in these studies. A left spinal cord hemisection at the second cervical segment was carried out as previously described^8^,^9^.

### Electromyography (EMG) analysis

In all rats immediately after C2Hx performed on day 0, paralysis of the ipsilateral hemidiaphragm was confirmed by EMG analysis as previously described^9^. Seven days after nanoconjugate injection each rat underwent a second EMG to analyze the muscle function.

### Nanoconjugate injections

Immediately after the C2Hx and the EMG recording on day 0, all rats received left intradiaphragmatic injections of one of three solutions; WGA-HRP-Au-pro-THP (n = 5), WGA-HRP-Au-pro-DPCPX (n = 3), or WGA-HRP-Au (n = 3). The solutions were injected in 10-microliter increments using a Hamilton syringe (cat. 7637-01; 7803-02). The total amount of the injected nanoconjugate was standardized based on animal’s weight and dilution of the stock solution (WGA-HRP-AuNP-pro-THP 0.06 mg/kg; WGA-HRP-AuNP-pro-DPCPX 0.15 μg/kg).

In a previous study THP and DPCPX were administered systemically (intravenous)^10^. Due to the intradiaphragmatic injection used in this study, a dose response study was analyzed to determine the lowest dose with maximal recovery. Since the injections were targeted, due to the injection site and the WGA component, instead of global, the resulting dose injected that induced recovery of the diaphragm muscle was a fraction of the dose needed for systemic administration.

### Immunohistochemistry

Three days after intradiaphragmatic injection of WGA-HRP-AuNP-pro-THP, the rats (n = 2) were anesthetized and underwent transcardial perfusion with heparinized saline followed by 4% formaldehyde (Fisher, F-79). The cervical spinal cord segments 3–6 were removed and an insect pin was inserted to distinguish right from left. The medulla was removed and an insect pin was inserted. The tissue samples were post-fixed in 4% formaldehyde (24 h), and transferred to 30% sucrose for cryo-protection. Sections were cut transversely on a cryostat (50 μm) and collected in PBS (pH = 7.4).

The tissue sections were washed 3 times in immuno buffer (PBS + 0.3% triton) and then blocked using 10% normal horse serum (Gibco, Life Technologies) in immuno buffer. Sections were incubated in primary antibody goat anti-WGA (1:200, AS-2024, Vector Labs, Burlingame, Ca) diluted in 10% normal horse serum-immuno buffer solution (72 h). Sections were washed in Tris-PBS (Strack and Loewy, 1990), followed by incubation in the biotinylated secondary antibody, donkey anti-goat (1:400, Jackson Immunoresearch) overnight. Sections were washed in Tris-PBS. Sections were incubated in streptavidin tagged-Cy3 (1:1000, Jackson ImmunoResearch) for 4 hours in the dark. Sections were washed in Tris-PBS then mounted wet on slides. The sections were left to dry (approximately 20 min) several drops of buffered glycerol were dispensed onto the tissue sections and was immediately covered with the coverslip. Nail polish was applied to the edges to secure the coverslip and to prevent drying of the tissue.

The images were acquired using a Zeiss Axioimager.M2 fluorescent microscope. Images were taken using the red filter. Images were analyzed using the Zen 2 Blue edition software.

## Additional Information

**How to cite this article**: Zhang, Y. *et al*. Transporter protein and drug-conjugated gold nanoparticles capable of bypassing the blood-brain barrier. *Sci. Rep.* **6**, 25794; doi: 10.1038/srep25794 (2016).

## Supplementary Material

Supplementary Information

## Figures and Tables

**Figure 1:**
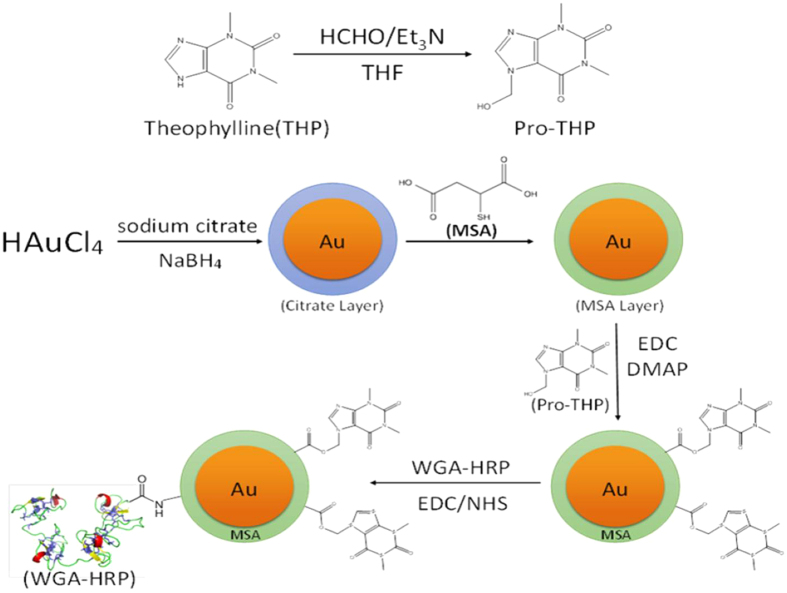
Synthesis schematic of transporter protein WGA-HRP and drug THP conjugated to the AuNPs. HCHO/Et_3_N; formaldehyde/triethylamine; THF, Tetrahydrofuran; HAuCl4, chloroauric acid; NaBH_4_, Sodium borohydride; Au, gold; MSA, mercaptosuccinic acid; EDC, 1-Ethyl-3-[3-dimethylaminopropyl]carbodiimide hydrochloride; DMAP, 4-Dimethylaminopyridine; WGA-HRP, wheat germ agglutinin-horseradish peroxidase; NHS, N-Hydroxysuccinimide.

**Figure 2:**
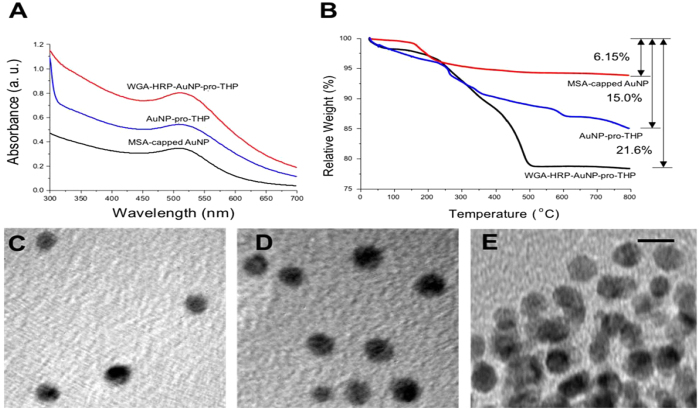
Characterization of transporter protein and drug-conjugated AuNPs. (**A**) UV-*vis* spectra of MSA-capped AuNP, AuNP-pro-THP, and WGA-HRP-AuNP-pro-THP in deionized water (pH = 5.8) at room temperature. The spectra are shifted along the vertical axis for easy comparison. (**B**) TGA data of weight loss for MSA-AuNPs of 6.15% (AuNP : MSA = 100 : 6.6), AuNP-pro-THP of 15.0% (AuNP : MSA : pro-THP = 100 : 6.6 : 11.1) and WGA-HRP-AuNP-pro-THP of 21.6% (AuNP : MSA : pro-THP : WGA-HRP = 100 : 6.6 : 11.1 : 11.5). (**C**) TEM image of MSA-capped AuNP. (**D**) TEM image of AuNP-pro-THP. (**E**) TEM image of WGA-HRP-AuNP-pro-THP. The scale bar for all TEM images is 5 nm.

**Figure 3:**
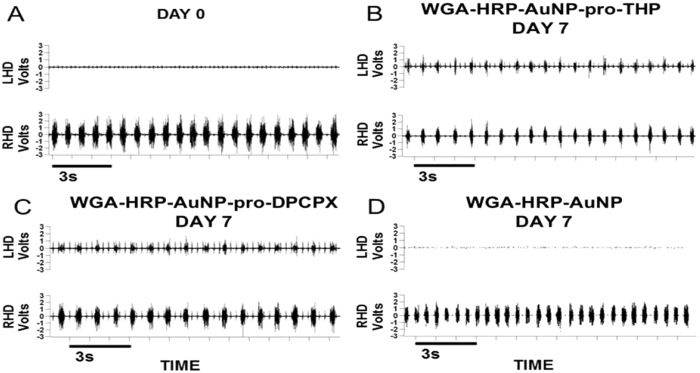
Electromyographic recording of the left (LHD) and right (RHD) hemidiaphragm obtained immediately after the left C2Hx confirming LHD paralysis (panel **A**), and 7 days after intradiaphragmatic administration of WGA-HRP-AuNP-pro-THP nanoconjugate 0.06 mg/kg (panel **B**), WGA-HRP-AuNP-pro-DPCPX nanoconjugate 0.15 μg/kg (panel **C**), or control nanoconjugate WGA-HRP-AuNP (panel **D**). Every breath is represented by one burst. Note (panel **A**) only displays bursting of the RHD similar to the control in (panel **D**). Note in (panels **B** and **C**) the LHD has a return of function demonstrated by the bursting pattern that is synchronous with the RHD.

**Figure 4:**
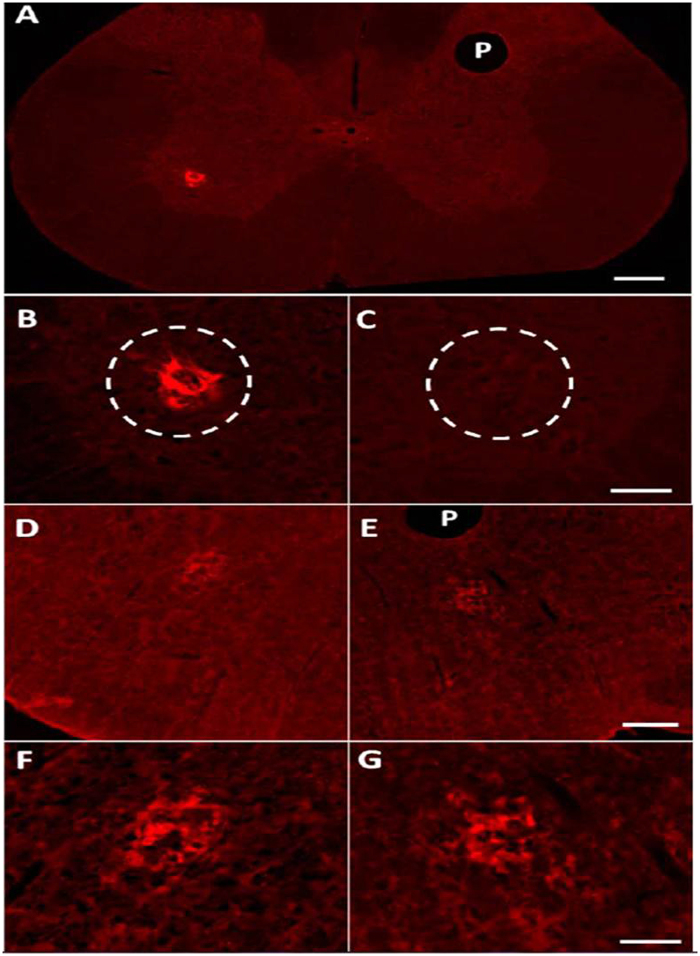
(**A**) Transverse section of the cervical spinal cord displaying ipsilateral anti-WGA positive labeling in the phrenic nuclei (PN) following intradiaphragmatic injection of the WGA-HRP-AuNP-pro-THP nanoconjugate. There is a lack of labeling in the contralateral PN. Scale bar is 200 μm, P notes pinhole to mark side contralateral to the injection. (**B**) Higher magnification of the ipsilateral PN shown in A displaying fluorescence from the anti-WGA antibody. (**C**) Higher magnification of the contralateral PN shown in (**A**)with a complete lack of WGA label. Scale bar = 50 μm. (**D,E**) Transverse sections of the medulla at the level of the rVRGs from the same rat shown in A. (**D**) Ipsilateral rVRG displaying fluorescence from the anti-WGA antibody. (**E**) Contralateral rVRG displaying fluorescence from the WGA antibody. P notes pinhole to mark side contralateral to the injection. Scale bar = 200 μm. (**F**) Higher magnification of the ipsilateral rVRG show in (**D**). (**G**) Higher magnification of the contralateral rVRG shown in E. Scale bar = 50 μm.
