# Supplementary material for: Transporter protein and drug-conjugated gold nanoparticles capable of bypassing the blood-brain barrier
Source: Sci Rep. 2016 May 16;6:25794. doi: 10.1038/srep25794 (PMC4867579; doi:10.1038/srep25794)

Supplemental Information

Transporter protein and drug-conjugated gold nanoparticles capable of bypassing the blood-brain barrier

Yanhua Zhang[1], Janelle Buttry Walker[2], Zeljka Minic[2], Fangchao Liu[1], Harry Goshgarian[2,] Guangzhao Mao[1,*]*

[1]Department of Chemical Engineering and Materials Science, Wayne State University, 5050 Anthony Wayne Drive, Detroit, MI 48202

[2]Department of Anatomy and Cell Biology, Wayne State University, 540 E Canfield St, Detroit, MI 48201

[*]Corresponding authors: gzmao@eng.wayne.edu and hgoshgar@med.wayne.edu

1. Nanoconjugate chemical composition calculations

Calculations of nanoconjugate drug and protein loading amounts based on TGA and TEM results are shown below.

The weight of one molar AuNP (W) with a diameter (D = 4 nm based on the TEM measurements) was estimated using the following formula using the bulk density of gold (ρ = 19.3 g/cm^3):

$$W = \rho \times \frac{1}{6}\pi D^3 \times 6.022 \times 10^{23} = 19.3\frac{g}{cm^3} \times \frac{1}{6}\pi\left(4 \times 10^{-7}\,cm\right)^3 \times 6.022 \times 10^{23} = 3.89 \times 10^5\,g/mol.$$

The molecular weight of MSA ($C_4H_6O_4S$) and pro-THP are 150 g/mol and 210 g/mol, respectively. The weight loss, 100:6.6 = AuNP:MSA, corresponds to 171 MSA molecules per AuNP as calculated below. As a result, each MSA-AuNP has 342 maximum reactive sites.

$$\text{No. of MSA molecules} = \frac{6.6}{150} \times \frac{3.89 \times 10^5}{100} = 171.16 .$$

The area of MSA coverage on AuNP (*A*) is calculated below:

$$A = \frac{\pi D^2}{\text{No. of MSA molecules}} = \frac{\pi (4nm)^2}{171.16} = 0.294 nm^2 = 29.4 \dot{\text{A}}^2 .$$

The weight loss of 15.0% (AuNP : MSA : pro-THP = 100 : 6.6 : 11.1) shows that 205.6 pro-THP molecules are conjugated to one AuNP as calculated below:

$$\text{No. of pro-THP molecules} = \frac{11.1}{210} \times \frac{3.89 \times 10^5}{100} = 205.6 .$$

This means that 60% of all possible reactive sites provided by MSA are occupied by pro-THP.

The total weight loss for the three-part nanoconjugate is 21.6% (AuNP : MSA : pro-THP : WGA-HRP = 100 : 6.6 : 11.1 : 11.5) indicates that each WGA-HRP is conjugated to 2 AuNPs as calculated below:

$$\text{No. of WGA-HRP molecules} = \frac{11.5}{22367} \times \frac{3.89 \times 10^5}{100} \approx 2 .$$

For pro-DPCPX drug (TGA spectra shown in Supplementary Fig. 4), the weight loss of 12.9% (AuNP : MSA : pro-DPCPX = 100 : 6.6 : 8.2) corresponds to 96 pro-DPCPX molecules conjugated to one MSA-capped AuNP. This means that 28% of MSA reactive sites are occupied by pro-DPCPX as calculated below:

$$\text{No. of pro-DPCPX molecules} = \frac{8.2}{332} \times \frac{3.89 \times 10^5}{100} = 96$$
.

The total weight loss for the three-part nanoconjugate is 21.0% (AuNP : MSA : pro-DPCPX : WGA-HRP = 100 : 6.6 : 8.2 : 11.8) indicates that each WGA-HRP is conjugated to 2 AuNPs as calculated below:

$$\text{No. of WGA-HRP molecules} = \frac{11.8}{22367} \times \frac{3.89 \times 10^5}{100} \approx 2.$$

2. Synthesis and characterization of DPCPX three-part nanoparticles

The synthesis steps of the three-part DPCPX nanoconjugate are similar to those of THP as illustrated in Fig. 1. DPCPX was converted to its pro-drug form, pro-DPCPX, using the Mannich reaction in order to conjugate DPCPX to MSA-capped AuNP with an ester bond linkage. The ester bond enables the hydrolytic release of DPCPX from its nano-carrier. In a typical reaction, 100 mg DPCPX was added to 1 ml 37% formaldehyde (in water), followed by the addition of 1.5 ml Et_3N with vigorous stirring. After the mixture became homogeneous, 2 ml THF was added. The reaction proceeded for 2 days in quiescent conditions to allow crystallization of pro-DPCPX. The pro-DPCPX crystals were dried and then dissolved in 20 mL DMSO to reach a final concentration of 5 mg/ml.

MSA-capped AuNP was concentrated to 5 mg(gold)/ml in 5 ml 0.1 M MES buffer. The pH value was adjusted to 4.7 using 0.1 M MES buffer to favor the ester bond formation. MES was used instead of acetic acid because acetic acid can react with the solvent. One ml of 5 mg/ml pro-DPCPX in DMSO solution was added to 5 ml DMSO containing 4.8 mg EDC and 1.7 ml of DMAP to start the Steglich Reaction. After 40 h reaction, the AuNP-pro-DPCPX product was washed in deionized water and concentrated several times.

In order to conjugate WGA-HRP to the above product through an amide bond to make the final nanoconjugate, the solution of the above product was diluted to 0.1 mg/ml (pro-DPCPX concentration) by deionized water and was maintained at 4°C and pH of 6.6. 5 mg WGA-HRP, 10 mg EDC, and 3 mg NHS were added to the solution. The reaction proceeded for 1 h followed by washing with deionized water. The concentration of the final stock was diluted to 0.5 mg (pro-DPCPX)/ml based on the pro-DPCPX input amount, the actual concentration was 0.14 mg/mL, which was determined by TGA analysis. The stock was diluted 50, 100, 150, and 300 times, corresponding to 2.8, 1.4, 0.93, and 0.46 μg (pro-DPCPX)/ml for the *in vivo* experiments. The nanoconjugate solutions can be stored at 4°C up to one month before *in vivo* experiments.

The Zetasizer and TGA results for pro-DPCPX synthesis products are located in SI Fig. 2B and SI Fig. 4, respectively. The hydrodynamic diameters measured by the zetasizer are 13.5 nm (zeta potential = -37 mV) for pro-DPCPX-AuNP and 32.7 nm (zeta potential = -27.4 mV) for WGA-HRP-AuNP-pro-DPCPX. The zetasizer measurements were conducted in PBS buffer (pH = 7.4) at 37°C.

3. Supplementary information figures

SI Figure 1. ^{1}H NMR spectra of A) THP and B) pro-THP. Both THP and pro-THP were dissolved in $CDCl_3$ at room temperature and were analyzed using a Merck 400MHz instrument. ^{1}H NMR δ 5.6 (broad s, 3 CH_2-OH) peak occurred in product confirming the formation of pro-THP.

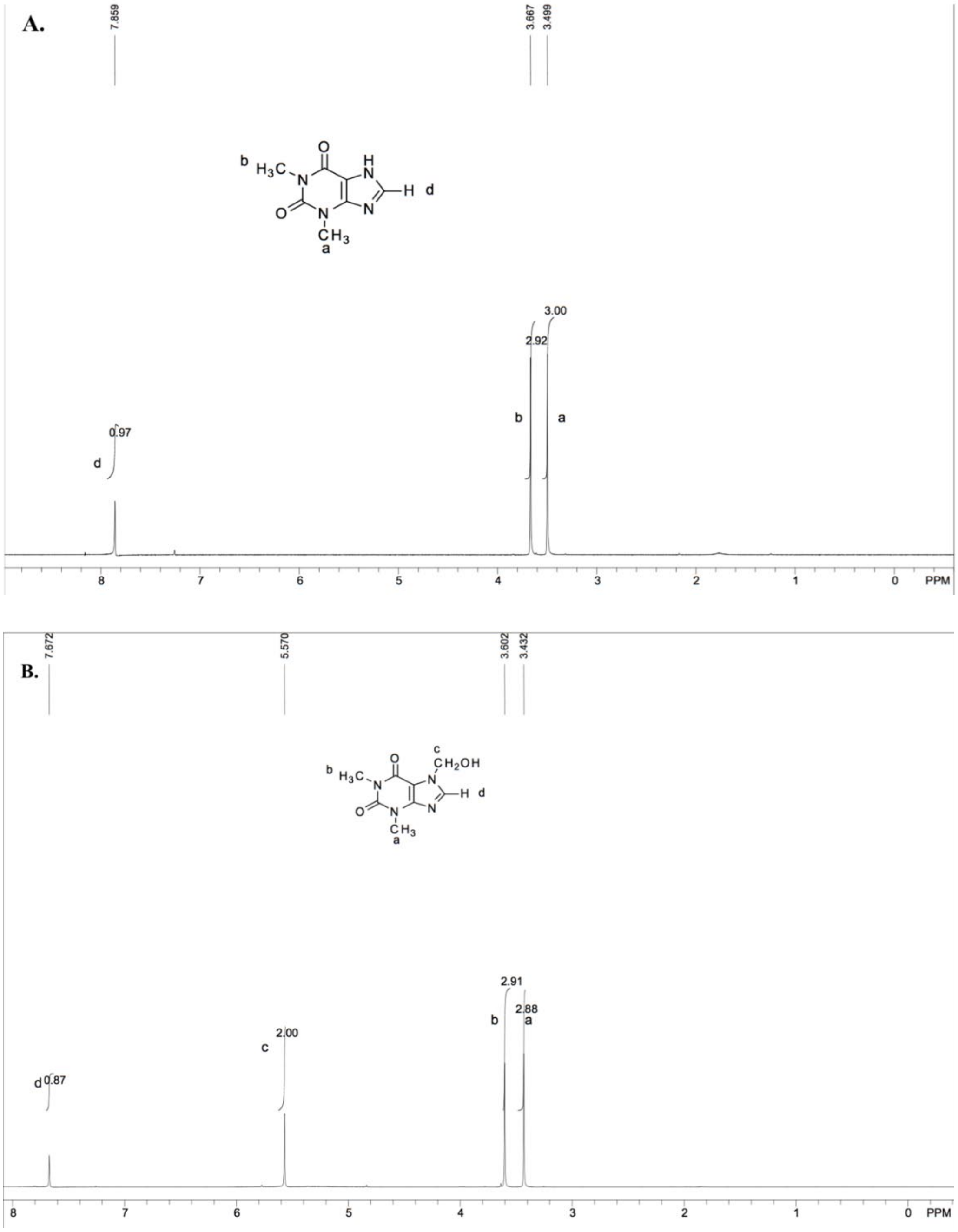

SI Figure 2. Zetasizer measurements of A) pro-THP nanoconjugates and B) pro-DPCPX nanoconjugates.

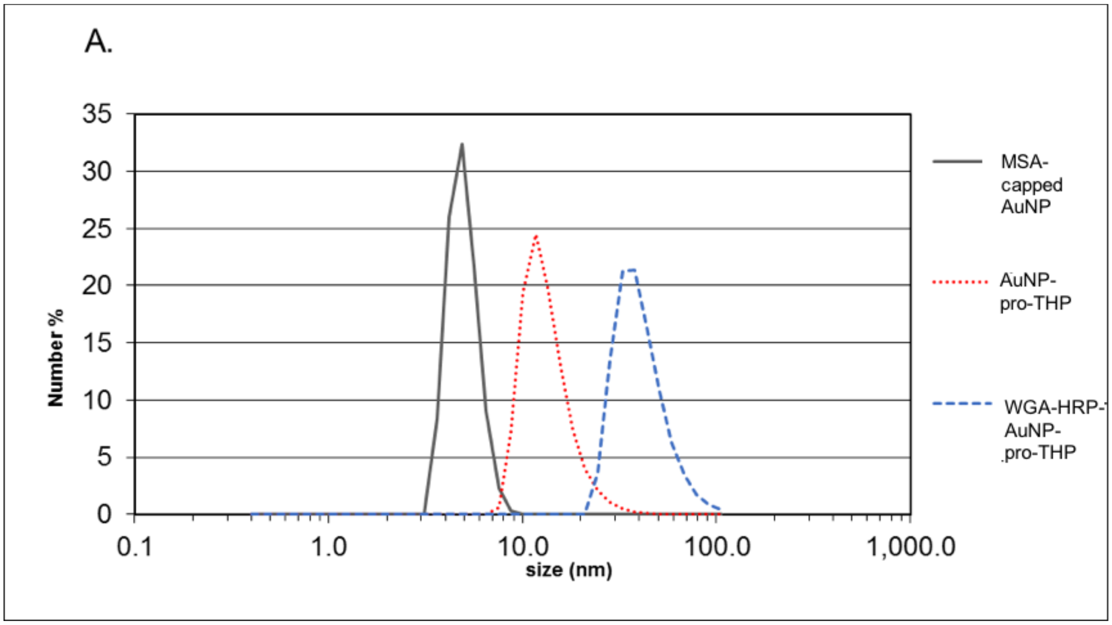
A.
35
30
25
20
15
10
5
0
Number %
0.1
1.0
10.0
100.0
1,000.0
size (nm)
MSA-capped AuNP
AuNP-pro-THP
WGA-HRP-AuNP-pro-THP

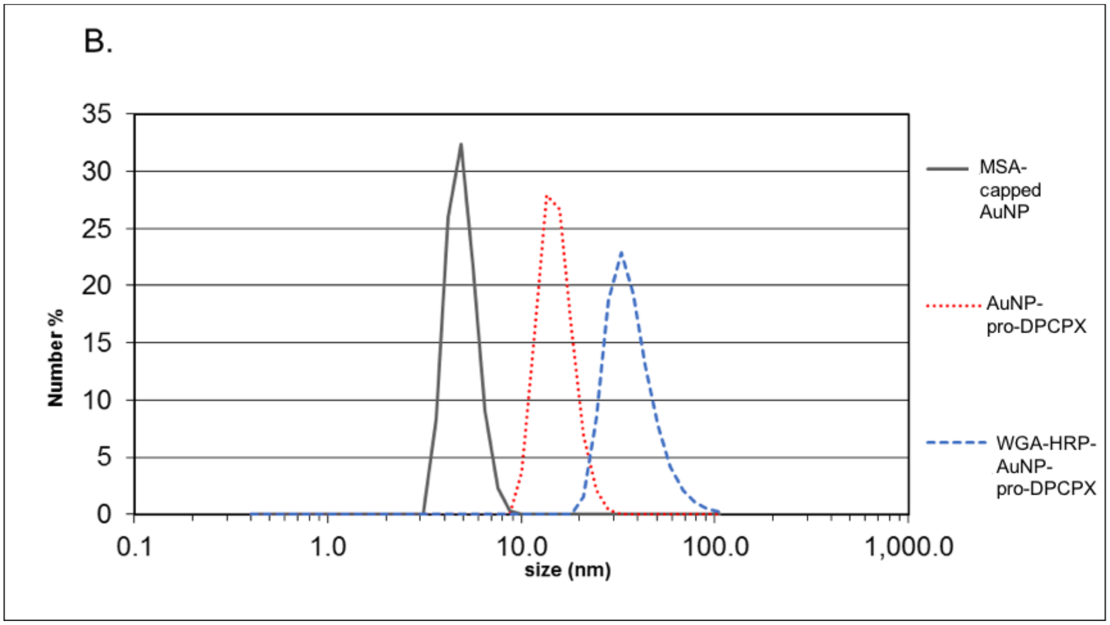
B.
35
30
25
20
15
10
5
0
Number %
0.1
1.0
10.0
100.0
1,000.0
size (nm)
MSA-capped AuNP
AuNP-pro-DPCPX
WGA-HRP-AuNP-pro-DPCPX

SI. Figure 3. TEM size distribution histograms of the AuNP component of the nanoconjugate after each step of synthesis; A) MSA-capped AuNP, B) AuNP-pro-THP, and C) WGA-HRP-AuNP-pro-THP.

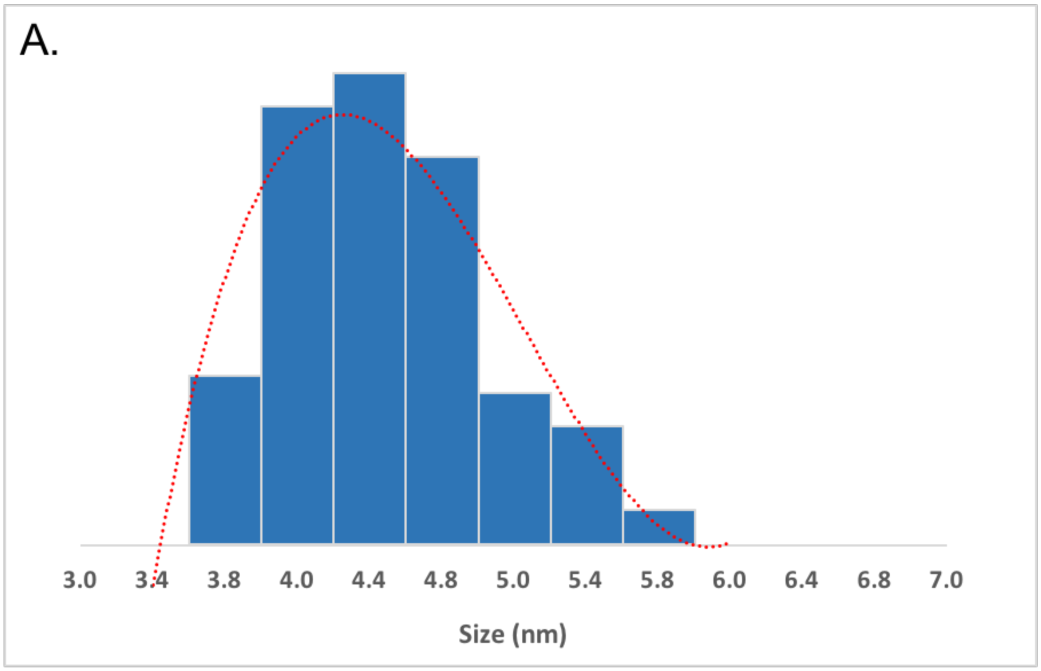

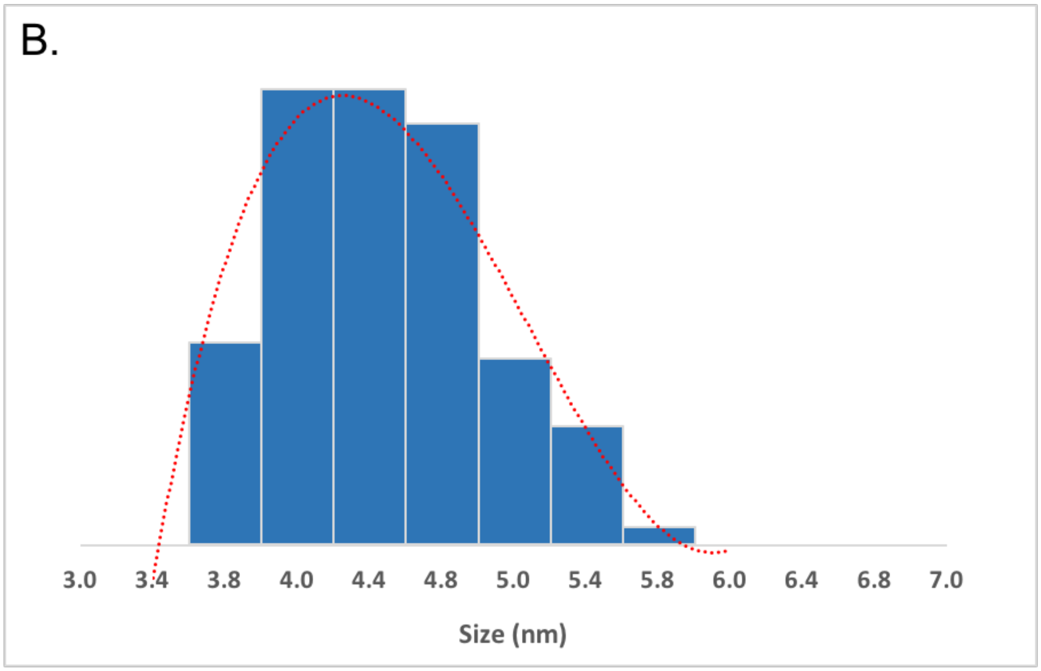

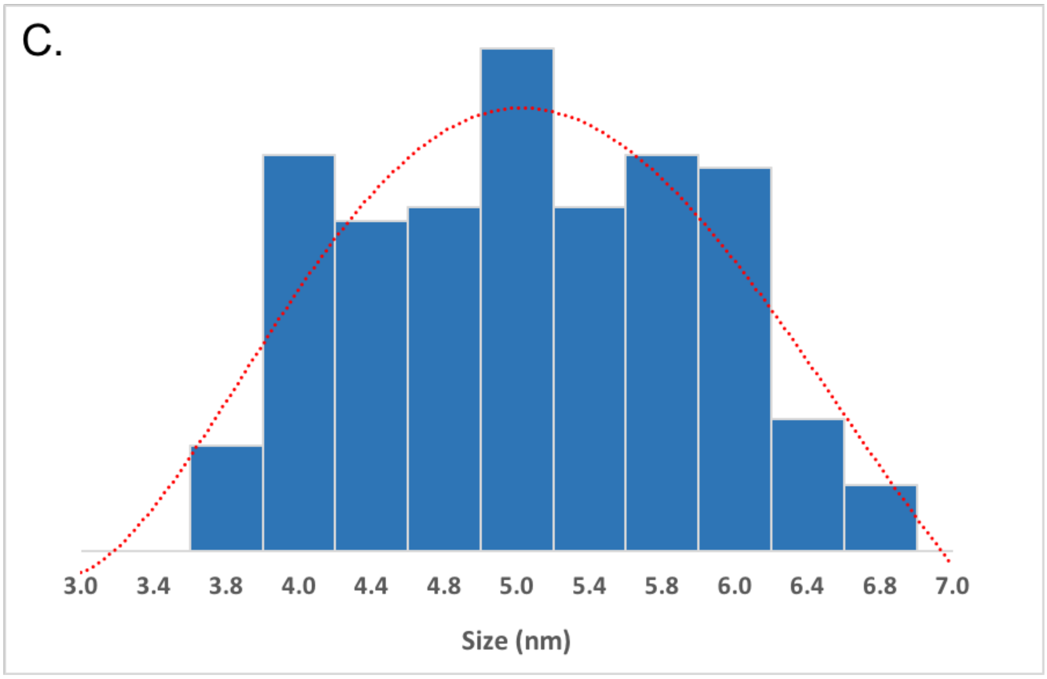

C.
3.0
3.4
3.8
4.0
4.4
4.8
5.0
5.4
5.8
6.0
6.4
6.8
7.0
Size (nm)

SI. Figure 4. TGA spectra of pro-DPCPX nanoconjugates.

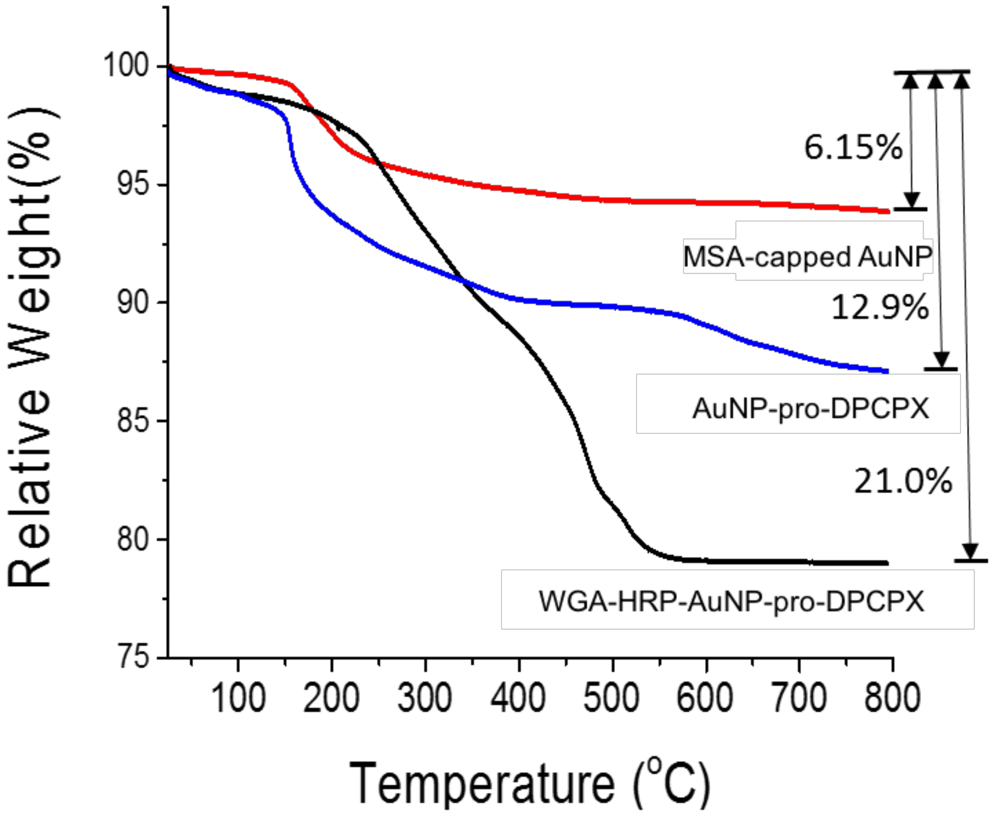

SI. Figure 5. MS spectra of supernatant after 12 h of the AuNP-pro-THP nanoconjugate in A) ACSF or B) HEPES buffer, and C) control pro-THP alone dissolved in HEPES.

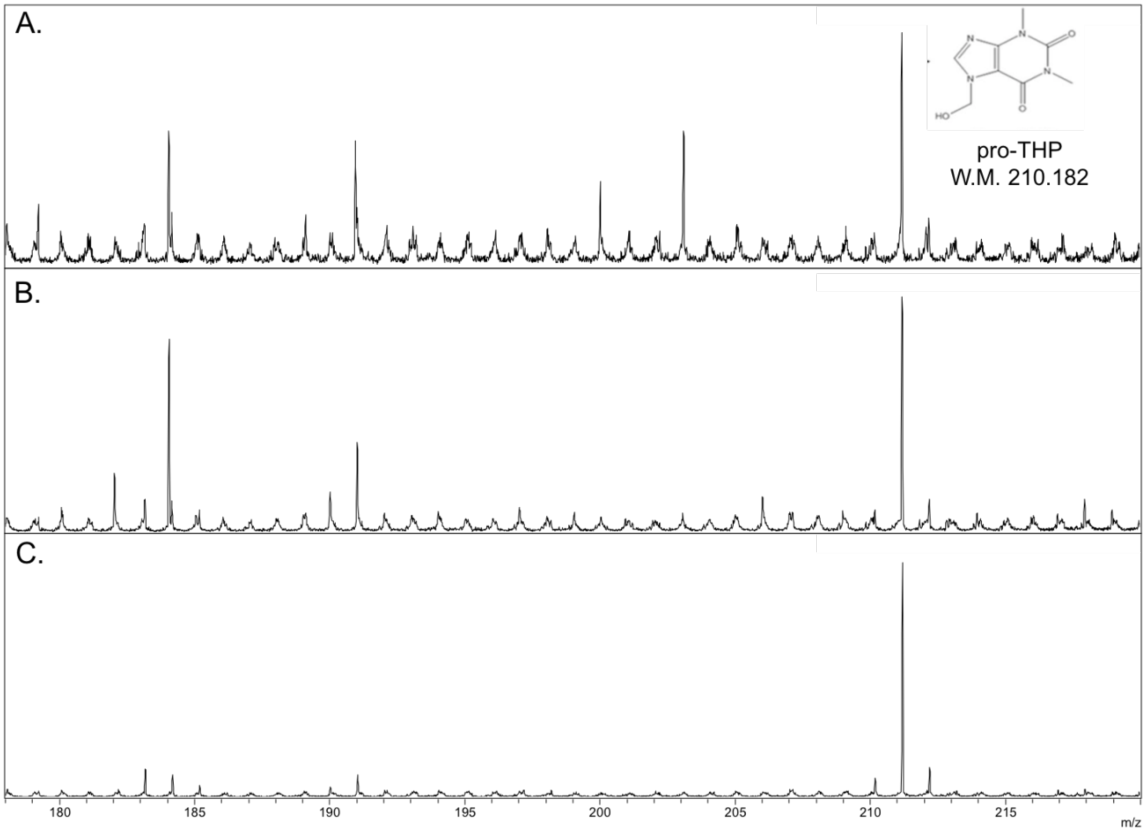

Supplement: Supplementary Information [file srep25794-s1.pdf]
